# Delirium as an Atypical Presentation of Severe Aortic Stenosis in a Cognitively Intact Nonagenarian With Atrial Fibrillation: A Case Report

**DOI:** 10.7759/cureus.93623

**Published:** 2025-10-01

**Authors:** Ishaque Rafai, Sana Javed Malik, Ariba Ali

**Affiliations:** 1 Internal Medicine, Queen Alexandra Hospital, Portsmouth, GBR; 2 Acute Medical Unit, Queen Alexandra Hospital, Portsmouth, GBR; 3 Medicine and Surgery, Queen Alexandra Hospital, Portsmouth, GBR

**Keywords:** aortic stenosis, delirium, diuresis, elderly, elevation, geriatric medicine, hypoperfusion, nt-probnp, troponin

## Abstract

Delirium is a common, multifactorial neuropsychiatric syndrome, especially prevalent among older adults. While infection, metabolic disturbances, and medication effects are frequent causes, cardiac etiologies, particularly cerebral hypoperfusion secondary to valvular heart disease, may be under-recognized. We report the case of a 90-year-old woman with severe aortic stenosis and chronic atrial fibrillation who presented with acute delirium despite preserved baseline cognition. The initial evaluation excluded common precipitants of delirium, including infection, metabolic derangements, polypharmacy, and structural central nervous system pathology. Investigations revealed evidence of acute decompensated heart failure, including volume overload, elevated N-terminal pro B-type natriuretic peptide (NT-proBNP), and a severely stenotic aortic valve on transthoracic echocardiogram (TTE). Conservative treatment with intravenous diuretics led to the rapid resolution of delirium within 72 hours. This case highlights an atypical presentation of severe aortic stenosis and underscores the importance of considering cardiac causes in elderly patients presenting with delirium in the absence of other identifiable factors.

## Introduction

Aortic stenosis (AS) is the most prevalent valvular heart disease affecting the elderly population, with a prevalence of up to 10% in individuals aged 80 years and older [1]. The typical clinical presentation includes exertional dyspnea, syncope, or angina. However, in frail or very elderly patients, presentations can be atypical, and subtle signs of decompensation may be missed [2]. Delirium is an acute disturbance in attention and cognition, occurring in up to 50% of hospitalized older adults [3]. Common precipitants include infection, metabolic imbalances, central nervous system (CNS) pathology, and drug-related effects. Nevertheless, cerebral hypoperfusion due to low cardiac output states, such as severe aortic stenosis compounded by atrial fibrillation (AF), is an underappreciated cause of delirium.

This report describes a nonagenarian patient in whom acute delirium was the sole presenting feature of acute decompensated heart failure in the setting of severe AS and chronic AF. It emphasizes the necessity of a comprehensive evaluation and tailored management in elderly patients with delirium.

## Case presentation

A 90-year-old woman was brought to the emergency department with a three-day history of acute confusion, inattention, and agitation. She had no fever, urinary symptoms, recent medication changes, or falls. Her baseline cognition was well-preserved, demonstrated by an Abbreviated Mental Test Score (AMTS) of 10/10 two weeks prior [4]. On presentation, her AMTS had declined significantly to 4/10, indicating marked cognitive impairment. She lived independently with assistance, ambulated with a walker, and had a Clinical Frailty Score (CFS) of 5 [5], corresponding to mild frailty characterized by slowing and needing help with higher-order instrumental activities of daily living. Her care plan included a Do Not Attempt Resuscitation (DNAR) order.

Her medical history was notable for severe calcific aortic stenosis, with an aortic valve area (AVA) of 0.6 cm² and a gradient of 60 mmHg on transthoracic echocardiography six months earlier. She also had chronic AF managed with sotalol and apixaban, chronic kidney disease stage III, a prior transient ischemic attack (TIA), and a history of pulmonary embolism on long-term anticoagulation. Her regular medications included diclofenac gel, Adcal D3 (calcium carbonate and vitamin D3) twice daily, macrogol once daily, bisoprolol 2.5 mg once daily, apixaban 5 mg twice daily, and lansoprazole 15 mg once daily.

Upon presentation, her vital signs were as follows: blood pressure 130/60 mmHg, heart rate 68 beats per minute (with an irregularly irregular rhythm consistent with atrial fibrillation), respiratory rate 18 breaths per minute, and oxygen saturation 96% while receiving 2 liters of oxygen for comfort. She was afebrile. Cardiovascular examination revealed an irregular rhythm and a grade 4/6 systolic ejection murmur at the right upper sternal border, with elevated jugular venous pressure. Respiratory examination revealed bibasal crepitations. Neurologically, she was disoriented to time and place and showed signs of inattention, but no focal neurological deficits were detected.

Differential diagnosis and workup for delirium

A comprehensive evaluation was performed to identify common causes of delirium. Laboratory tests included a complete blood count (CBC), C-reactive protein (CRP), urinalysis, chest radiography, electrolyte levels, glucose levels, liver function tests, calcium levels, magnesium levels, and a review of medications. The findings are summarized in Table 1.

**Table 1:** Laboratory findings with reference range

Parameters	Result	Reference Range
Hemoglobin (Hb)	107 g/L	115–150 g/L (women)
White Blood Cell Count (WBC)	6.2 x10⁹/L	4.0–11.0 x10⁹/L
Platelet Count	124 x10⁹/L	150–400 x10⁹/L
Red Blood Cell Count (RBC)	3.59 x10¹²/L	4.1–5.1 x10¹²/L (women)
Hematocrit (HCT)	0.331 L/L	0.37–0.47 (women)
Mean Corpuscular Volume (MCV)	92.2 fL	80–100 fL
Mean Corpuscular Hemoglobin (MCH)	29.9 pg	27–33 pg
Mean Corpuscular Hemoglobin Concentration	324 g/L	320–360 g/L
Red Cell Distribution Width (RDW)	14.4 %	11.5–14.5 %
Mean Platelet Volume (MPV)	9.9 fL	7.5–11.5 fL
Neutrophil Count	4.6 x10⁹/L	2.0–7.5 x10⁹/L
Lymphocyte Count	0.9 x10⁹/L	1.0–3.5 x10⁹/L
Monocyte Count	0.5 x10⁹/L	0.2–0.8 x10⁹/L
Eosinophil Count	0.1 x10⁹/L	0.0–0.4 x10⁹/L
Basophil Count	0 x10⁹/L	0.0–0.1 x10⁹/L
Urea	10.9 mmol/L	2.5–7.1 mmol/L
Sodium	142 mmol/L	135–145 mmol/L
Potassium	4.9 mmol/L	3.5–5.1 mmol/L
Creatinine	188 µmol/L	60–110 µmol/L
Estimated Glomerular Filtration Rate (eGFR)	35 mL/min/1.73 m²	>60 mL/min/1.73 m²
Phosphate	1.08 mmol/L	0.8–1.5 mmol/L
Calcium	2.23 mmol/L	2.15–2.55 mmol/L
Total Bilirubin	4 µmol/L	0–21 µmol/L
Total Protein	61 g/L	60–80 g/L
Albumin	33 g/L	35–50 g/L
Alkaline Phosphatase (ALP)	55 U/L	30–120 U/L
Alanine Aminotransferase (ALT)	9 U/L	7–56 U/L
Corrected Calcium (COCA)	2.34 mmol/L	2.15–2.55 mmol/L
C-Reactive Protein (CRP)	6 mg/L	<5 mg/L
NT-proBNP	9807 pg/mL	0–400 pg/mL
Troponin	216–220 ng/L	<14 ng/L
TSH	1.16 mIU/L	0.4–4.0 mIU/L
Folate	5.3 ng/mL	3.0–20.0 ng/mL
Vitamin B12	255 pg/mL	200–900 pg/mL

**Figure 1:**
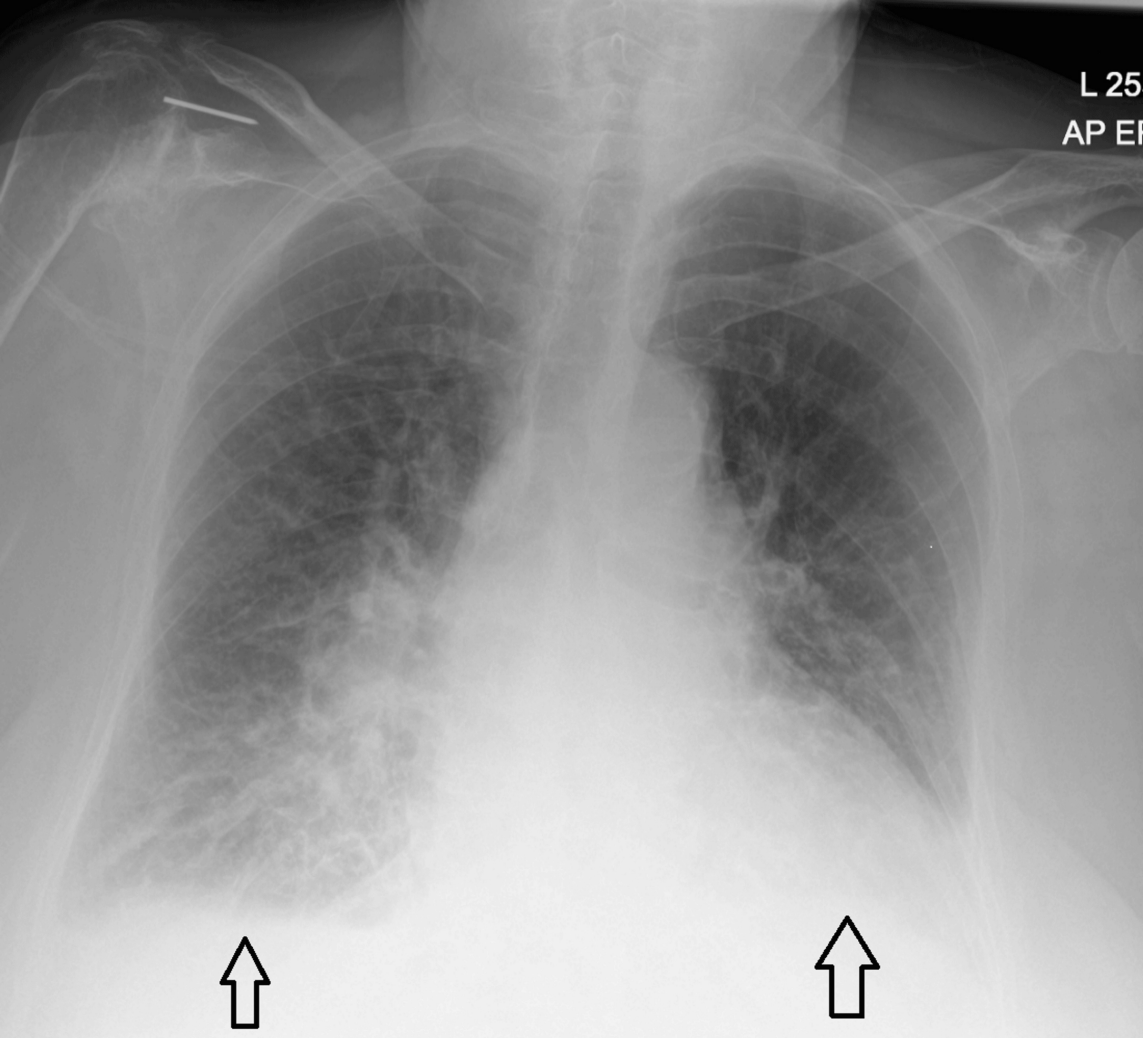
Chest X-ray on admission showing bilateral pulmonary congestion.

Infection was considered unlikely, given a normal white blood cell count and CRP, a clear urine dipstick, and no focal consolidation on chest X-ray (Figure 1). Metabolic causes were excluded as electrolytes and liver function tests were within expected ranges for her age and chronic kidney disease. A medication review revealed no recent changes or use of high-risk drugs. The neurological examination showed no new focal deficits, and a computed tomography (CT) scan of the head was considered unnecessary. She maintained adequate oxygenation on supplemental oxygen provided for comfort, with no evidence of hypoxia or hypercapnia, as indicated by the venous blood gas findings presented in Table 2.

**Table 2:** VBG findings VBG: venous blood gas

Parameter	Result	Normal Reference Range
pH	7.44	7.35–7.45
pO₂	6.4 kPa (48 mmHg)	4.6–6.0 kPa (venous blood)
pCO₂	5.5 kPa (41 mmHg)	4.7–6.0 kPa (venous blood)
Bicarbonate (HCO₃⁻)	27.6 mmol/L	22–26 mmol/L
Lactate	1.0 mmol/L	< 2.0 mmol/L

**Figure 2:**
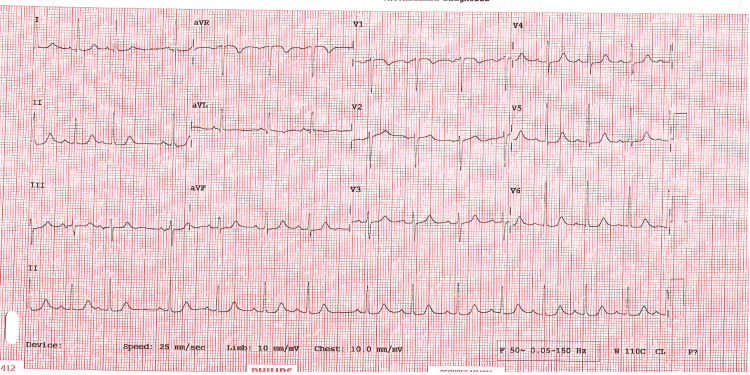
ECG showing no features of ischemia.

**Figure 3:**
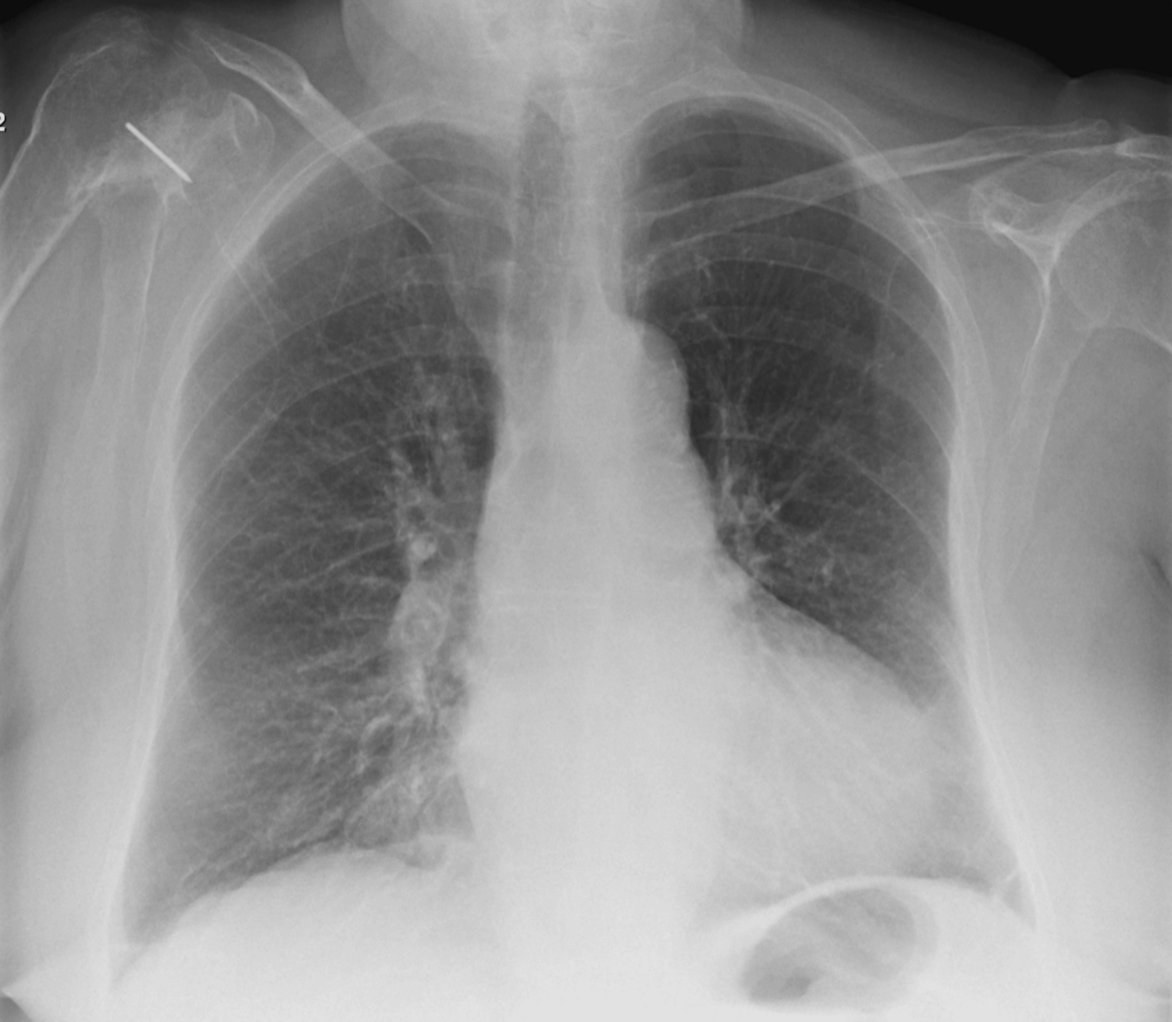
Chest X-ray three months prior to admission.

**Figure 4:**
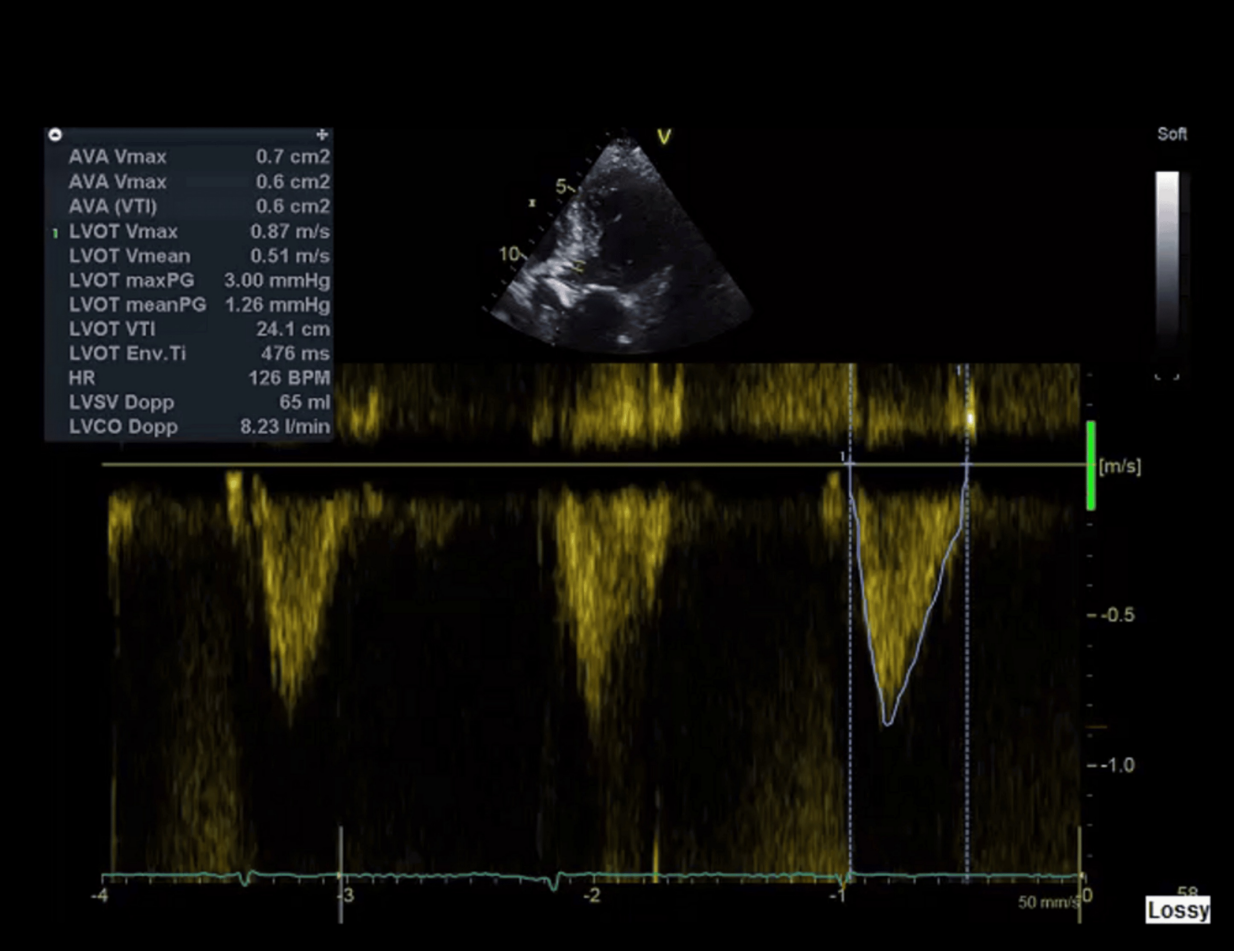
Echocardiograph showing indexed aortic valve area=0.6 cm².

**Figure 5:**
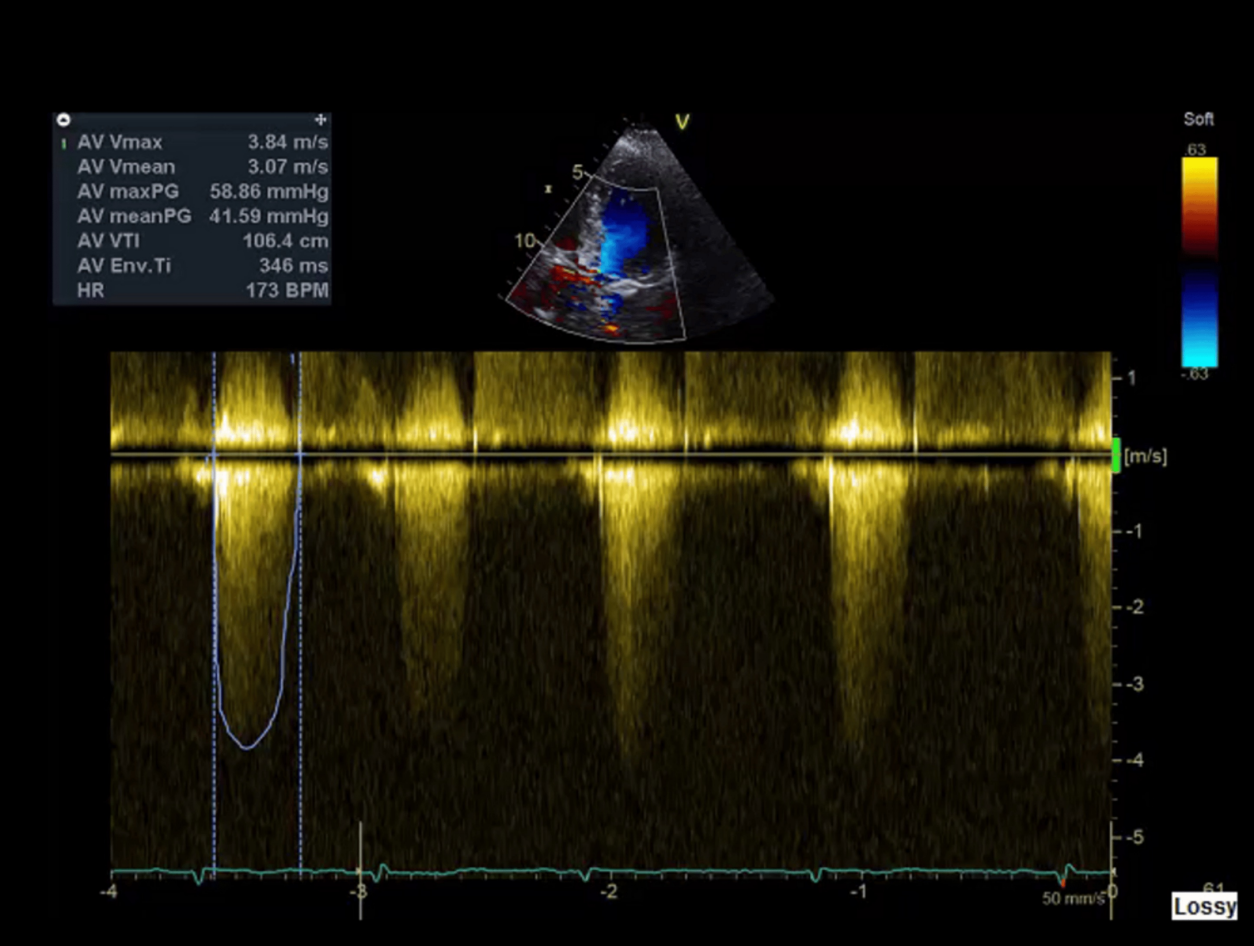
Echocardiograph showing a peak velocity of 3.84 m/s and a mean gradient of >40 mmHg.

Additionally, there was no evidence of pain, urinary retention, constipation, recent sleep deprivation, or dehydration. Cardiac evaluation revealed an elevated NT-proBNP of 9807 pg/mL and a mildly elevated troponin I of 220 ng/L (falling to 216 ng/L), consistent with type 2 myocardial injury rather than acute ischemia. The electrocardiogram (ECG) showed no signs of acute ischemia (Figure 2). The chest X-ray demonstrated bilateral pulmonary congestion (Figure 2), which was compared to the baseline chest X-ray taken three months prior (Figure 3). Transthoracic echocardiography showed severe aortic stenosis (AVA 0.6 cm², peak velocity 3.84 m/s) (Figures 4, 5).

Diagnosis

The diagnosis of delirium secondary to cerebral hypoperfusion from acute decompensated heart failure in the context of severe aortic stenosis and atrial fibrillation was established based on clinical and investigative findings.

Management and outcome

The patient was treated conservatively with intravenous furosemide 40 mg once daily for two days, followed by oral bumetanide 1 mg daily upon stabilization. Within 72 hours, her delirium completely resolved without the use of antipsychotics or sedatives. Pulmonary congestion improved clinically and radiologically, and no deterioration in renal function was noted.

She was discharged home at her baseline cognitive status. Outpatient follow-up was arranged with her general practitioner for renal function monitoring in two weeks. Cardiothoracic surgery consultation was not pursued due to her preferences for conservative management.

## Discussion

Delirium in older adults is often multifactorial, but low cardiac output states are frequently overlooked contributors [6,7]. Severe AS causes a fixed obstruction to blood flow through the left ventricular outflow tract (LVOT), significantly increasing afterload on the left ventricle. This obstruction limits the heart's ability to increase stroke volume and cardiac output, especially during periods of stress or increased metabolic demand. Moreover, delirium has been shown to predict worse outcomes in patients with acute heart failure admitted to intensive care units, underscoring the importance of recognizing and managing this complication promptly [8].

In patients with AF, the normal coordinated contraction of the atria is lost, resulting in the absence of the atrial kick. The atrial kick normally contributes up to 20-30% of ventricular filling during diastole, which is especially important in patients with stiff or hypertrophied ventricles, as seen in AS. Without this contribution, ventricular preload is reduced, impairing diastolic filling. Because the LVOT is fixed and noncompliant, cardiac output becomes highly dependent on adequate preload. The combination of fixed outflow obstruction and decreased preload (due to AF) leads to reduced stroke volume and diminished cardiac output, despite a preserved left ventricular ejection fraction (LVEF). This results in systemic hypoperfusion, including cerebral hypoperfusion. The brain, particularly in elderly or frail patients, is vulnerable to even mild reductions in perfusion. Inadequate cerebral blood flow can cause acute cognitive dysfunction, manifesting as delirium. Additionally, patients with severe AS are often preload-sensitive and prone to hemodynamic instability, meaning small changes in volume status or rhythm can precipitate acute decompensation. Thus, the interplay of severe AS and AF creates a perfect storm for cerebral hypoperfusion and delirium, highlighting the need for clinicians to consider cardiogenic causes when evaluating acute cognitive changes in this population [6].

Elevated troponin, NT-proBNP, and pulmonary congestion in this patient supported the diagnosis of acute heart failure. The rapid resolution of delirium following diuretic treatment further reinforced the cardiac origin of her symptoms. The diagnosis of delirium was made based on the DSM-5 criteria, which include acute onset and fluctuating course of impaired attention and cognition, ensuring a systematic and standardized clinical assessment [9].

This case highlights the importance of systematically excluding common precipitants of delirium using standardized assessments. Tools such as the Delirium Etiology Checklist help rule out potential causes, including metabolic disturbances, infections, drug effects, hypoxia, and other factors, such as constipation, dehydration, and sleep deprivation. Additionally, the 4AT screening tool, which evaluates Alertness, the Abbreviated Mental Test (AMT4), Attention, and Acute change or fluctuating course, should be used in conjunction with clinical judgment to support accurate and timely diagnosis [3,10]. The absence of infection, medication triggers, CNS pathology, and metabolic disturbances in this patient pointed to a cardiogenic cause.

In frail, very elderly patients, invasive interventions for severe AS, such as valve replacement, may not be suitable. Both the 2020 American College of Cardiology/American Heart Association (ACC/AHA) guidelines [1] and the 2021 European Society of Cardiology/European Association for Cardio-Thoracic Surgery (ESC/EACTS) guidelines [2] recommend individualized treatment balancing life expectancy, frailty, symptom burden, and procedural risks. Diuretics remain the first-line therapy for symptomatic relief in such patients.

## Conclusions

Delirium may be the sole clinical manifestation of cardiac decompensation in elderly patients with severe AS and AF. A structured, systematic approach to ruling out common causes is essential. Clinicians must maintain a high index of suspicion for cardiogenic delirium and consider conservative management strategies when invasive interventions are contraindicated or undesired.
